# Changemakers as digital makers: Connecting and co-creating

**DOI:** 10.1007/s10639-022-10892-1

**Published:** 2022-01-24

**Authors:** Emma Whewell, Helen Caldwell, Mark Frydenberg, Diana Andone

**Affiliations:** 1 University of Northampton, University Drive, Northampton, NN1 5PH UK; 2 Computer Information Systems Department, Bentley University, Waltham, MA 02452 USA; 3 Politehnica University of Timisoara, Pta Victoriei No. 2, Timisoara, Romania

**Keywords:** Augmented and virtual reality, Twenty-first century abilities, Cooperative/collaborative learnings-cultural projects, Distributed learning environments

## Abstract

This paper presents data from two international projects focused on the interaction between changemaking and digital making in university students. The data is drawn from the contributions of 63 university students located in the United States, Romania, Spain, Belgium, Norway, Denmark and England. Using a design thinking methodology and a thematic analysis of student responses, the aim was to understand how the creative use of immersive technologies, such as augmented and virtual reality, might create an environment for changemaking practices in an international context. Findings suggest that students demonstrated not only enhanced digital skills and student engagement but increased cultural competence and global mindfulness. International digital collaboration can create conditions for students to develop changemaker attributes and identify as changemakers within the spheres of entrepreneurship and education, preparing them to be a force for change in the world.

## Introduction

Technology and digital pedagogies have had a profound effect on how individuals communicate and interact across entrepreneurship and education. The last year has seen unprecedented changes to work patterns across the world. As a result, increased remote working and online teaching are likely to remain as positive outcomes of Covid-19. Immersive technologies, including virtual and augmented reality, (VR and AR), create real world, sensory environments that can allow people to be anywhere virtually, to experience the world through the eyes of others, and to respond in ways that were previously not possible.

This paper contributes an innovative perspective on changemaker attributes through the lens of constructionism and connectivism. It adds to the field of knowledge by combining the elements of changemaking and digital making in an international context. Students utilise interactive and immersive technologies as a means for content creation and building digital skills. The study draws upon the findings of two international projects that used immersive technologies to foster university students’ preparedness for global citizenship through developing cultural competence, digital literacy and changemaker identity in educational and entrepreneurial contexts. It charts the development of these attributes among university students across seven countries as they collaborate, communicate and co-create immersive digital learning experiences with their international partners.

The ubiquity of mobile devices, the internet, and collaboration and communication tools has led to a resurgence in digital making, activities in which students create digital media and artefacts using digital technologies (Bosco et al., 2019; Loy & Canning, 2013). Through the process of digital making, students apply subject matter knowledge as they use technology to share their learning. By combining active learning with problem solving through digital making, students build skills and create a culture of changemakers driven by a shared purpose for collaboration and communication (Toivonen, 2013; Warnecke, 2015).

If we combine changemaking with digital making in this way, the resulting skill set is consistent with the transformative ambitions of the United Nations (UN) Sustainable Development Goal (SDG) 4.7, which promotes sustainable development and global citizenship, and highlights the importance of understanding cultural diversity in an increasingly globalised world. Cultural sensitivity and the ability to interact in culturally appropriate ways are recognised as important in today’s connected society (Hammer et al., 2003; Li, 2020). Dziedziewicz, Gajda, & Karwowski define cultural competence as “the skills needed to function effectively in interactions with people who differ from an individual linguistically or culturally” (Dziedziewicz et al., 2014, p. 32). Whewell et al. (2020) suggest that collaborating with people from different cultures builds creativity and breaks down barriers. Importantly, cultural competence links with the concept of changemaking, which similarly emphasises empathy, tolerance, and mutual respect.

Although developed independently, both projects used the design thinking method to structure their international collaborations. As the research advanced, they were able to identify the key competencies developed by students related to changemaking and digital making. These formed the basis of this single study, which combines data from both sets of participants to examine the impact of the projects on their changemaker identities and practices. By surveying sets of student responses from the two projects, this paper explores the following research question:

How can digital technologies help university students develop changemaker attributes and identities across educational and entrepreneurial contexts?

### Description of the projects

The data for this study is drawn from two projects: Digital Learning Across Boundaries: Developing Changemakers (DLAB) and TalkTech: An Exploration of Technology, Digital Media and Culture across Continents. Both are focused on social innovation, digital making, cultural competence and technology enhanced learning. However, DLAB looks at educational applications whereas TalkTech emphasises entrepreneurship and business applications.

Both projects focus upon immersive technologies in their use of VR and AR. VR can be described as the computer-generated simulation of a 3D environment via a headset or goggles, achieving a strong sense of being present in that virtual environment. Specialised headsets with high-resolution displays can provide high quality immersive experiences. Inexpensive VR viewers such as Google Cardboard enable users to experience VR content by inserting their smartphones into the viewer. AR applications bring new insights to real world objects and scenarios by using the built-in camera in a mobile device to scan an image, which then causes related multimedia content (often images, maps, hyperlinks, video, or text) to appear overlaid on the image. Some AR apps make use of a mobile device’s GPS (Global Positioning System) capabilities to provide location-relevant results.

Both projects use digital tools and strategies to nurture changemaker attributes such as self-confidence, innovation and creativity, critical thinking, empathy, reflection and communication. A particular emphasis is the communication strand, which aims for ‘high levels of digital literacy, cooperative learning and co-construction of meaning with others’ (Alden-Rivers, Armellini, Maxwell, et al., 2015a, p.11). The authors became aware of each other’s work in the use of immersive technologies to promote digital literacy, changemaking and cultural awareness, and considered the possibility that these common themes enable their students to develop changemaker attributes. This paper investigates how students participating in these projects develop changemaker attributes and identities that prepare them to make an impact in the world.

### The TalkTech project

TalkTech (Frydenberg & Andone, 2019) is a collaborative learning project that pairs students from digital technology courses in the United States and in Romania to work together each fall semester to debate current technologies and create digital media artefacts that develop computational thinking and digital literacy skills. Students negotiate which tools to use for communication and collaboration, as they create AR and VR experiences to share cultures across continents. Students are responsible for researching, selecting, and learning to use appropriate collaboration, communication, and digital media creation tools necessary to complete the project, which lasts approximately eight weeks during the fall semester. In the process, students learn how new technologies can be used in an entrepreneurial context, and find opportunities to applying new technologies as they work as members of international teams. Through their participation in the TalkTech project, students demonstrate self-awareness of their own abilities to use technology, develop confidence and communication skills, and show they can be flexible when working with their partners. Their projects give them the opportunity to express themselves creatively, as they create virtual worlds that until now could have only existed in their dreams. All these experiences demonstrate qualities of changemakers (Garrett, 2020) in the context of entrepreneurship.

Since 2017 Romanian and American students have worked in international teams to create and share original VR experiences. These include cultural or business landmarks such as tech retail shops, public art, coffee shops, local restaurants, sports venues, supermarkets, and the university campus (TalkTech: An Exploration of Technology, Digital Media, and Culture across Continents, 2021). The TalkTech project examines the process of creating original VR content to develop information technology literacy skills, while navigating cultural boundaries and exploring applications of VR in business as future entrepreneurs, especially in the areas of marketing and advertising.

While many VR tools allow users to experience content created by others, the CoSpaces platform (CoSpaces EDU: Make AR & VR In the Classroom, 2021) encourages students to become creators of content (Friedman & Mandelbaum, 2012). The instructors provided students with a short demonstration of CoSpaces, and after that, students were responsible to follow online tutorials or watch instructional videos as they became proficient in using the tool.

**Fig. 1:**
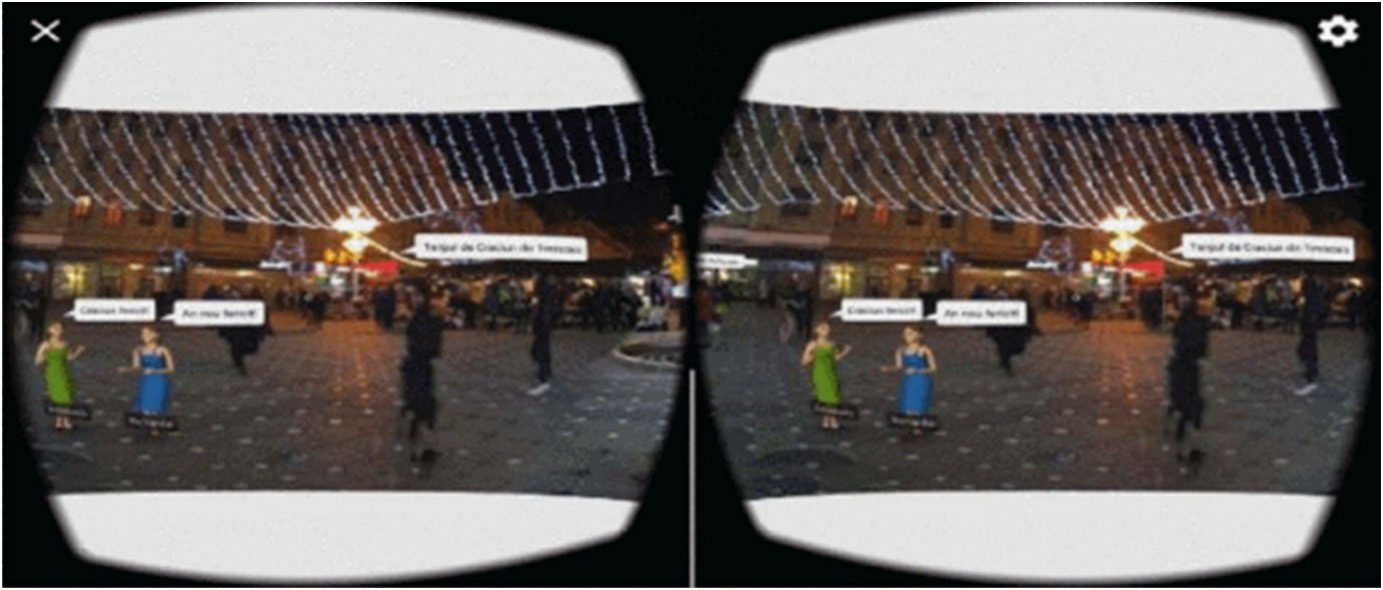
VR Evidence of Timisoara during the Winter Fair as viewed through Google Cardboard VR viewer in the TalkTech project

**Fig. 2:**
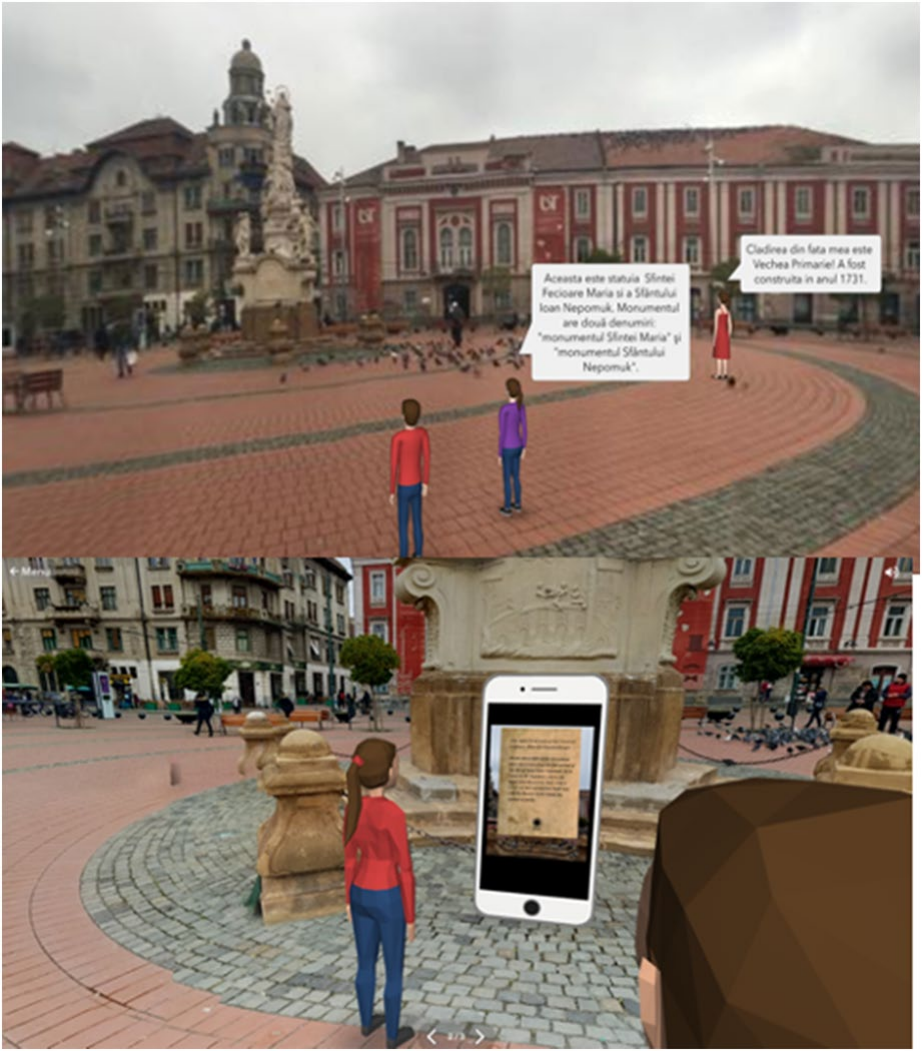
Liberty Square, Timisoara in Augmented reality and in Virtual reality, produced by a TalkTech international team of students

Students can combine a 360-degree image with 3D objects or characters and use a drag-and-drop visual programming language to animate them and add sounds to make it appear that they are interacting with each other. Figure 1 shows how the VR experience appears on a mobile device to be inserted into a Google Cardboard VR viewer. In this example, students created a VR experience in the old city centre of Timisoara, during the Winter Fair. Animated sprites provide a virtual tour of the Winter Fair and chat bubbles give additional information to the user. Figure 2 shows a visit to a Liberty Square in Timisoara in VR, which includes scanning a statue in AR to find out more information about it. After designing, developing, and coding their virtual worlds with the CoSpaces web application, students shared their worlds with others via a link or a QR code. They explored each other’s virtual worlds on their mobile devices using the CoSpaces mobile app using AR or VR views.

### The DLAB project

**Fig. 3:**
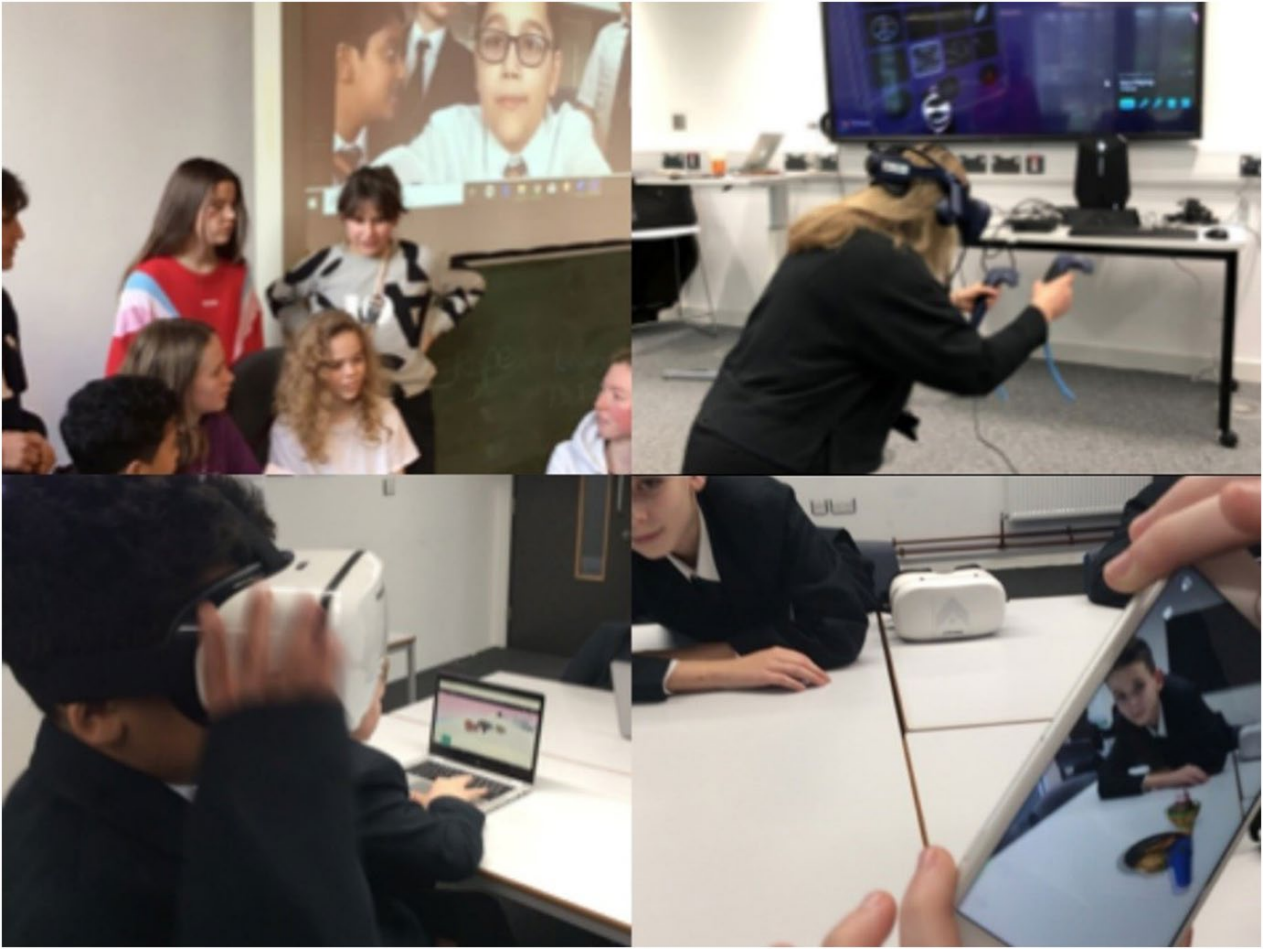
Use of CoSpaces, video conferencing and HTC Vives to develop and share digital artefacts

The Digital Learning Across Boundaries: Developing Changemakers project is a three-year Erasmus+ funded project, currently in its second year. The project uses immersive technologies, such as AR and VR, to blend physical and digital learning environments and provide creative opportunities for international collaboration among university student teachers, teachers and lecturers in England, Denmark, Norway, Belgium and Spain. Each year the project aims to use technology to find innovative solutions to identified problems by crossing physical, personal and environmental boundaries. Participants in the first year identified the social need to combat the association between video gaming and physical inactivity. They chose to address this issue by developing exergames using a range of technologies. Supported by their teachers and lecturers, the university students facilitated school pupils’ engagement with immersive technologies. During designated ‘international days’ they used videoconferencing and Twitter to communicate with their international partners and virtually visit each other’s classrooms. Figure 3 demonstrates how pupils used immersive technologies to share digital artefacts such as their favorite meal via CoSpaces and developed prototype exergames using the HTC Vives.

Participants developed their digital skills to be creative with AR and VR in an educational context, beginning by swapping 360° video and stills to gain immersive experiences of each other’s spaces and swapping artefacts such as digital meals designed in CoSpaces or animated AR Gif greetings. They went on to combine a range of apps and tools to invent exergames for each other to experience in AR and VR. These included VR basketball coaching, VR table tennis, combining VR with balance boards and challenging partners to explore an environment with the Cardboard Camera app. In prototyping and evaluating their invented exergames across the partner countries, participants gained a sense of having an impact and began to see themselves as changemakers.

## Review of literature

This section evaluates uses of immersive technologies, examines the changemaker movement and identifies the learning theories that provide a conceptual framework for the projects.

### Immersive technologies

Recent availability of AR apps for mobile devices and VR headsets for consumers have brought about a collision of augmented, virtual, and real worlds. Immersive technologies including augmented and virtual reality have brought about new forms of engagement across a range of industries in recent years alongside advances in mobile technologies (Abulrub et al., 2011). Healthcare, education, training and marketing are among the many industries that have integrated AR and VR into their daily experience, allowing people to create and experience digital environments that would otherwise be impossible.

Similarly, the use of VR in education to create collaborative learning experiences has progressively increased in recent years, as researchers explore its features, advantages, and limitations (Cortiz & Silva, 2017). Now more affordable, Oculus Rift, Oculus Go, HTC Vive, and Microsoft’s HoloLens headsets are finding their way into classrooms. As 5G technology becomes more available, the potential of these tools for learning will only increase (Bacca et al., 2014). The main uses of VR have been for exploring topics in depth through experiential learning, and it is noted that it often leads to increased engagement (Vasilevski & Birt, 2020). The wide availability of off-the-shelf VR content from providers such as Google (Cardboard, Earth, Street View, Expeditions, and Tour Creator) (Google VR, n.d.) allow pupils to engage with aspects of world culture, geography, or natural sciences in immersive learning environments. Teachers have incorporated simple VR experiences into their lessons such as virtual field trips, immersive games, and 3D painting (So & Lu, 2019). VR experiences have also provided realistic training environments in engineering classrooms. For example, Rogers et al. (2018) describe the effective use of VR for training pupils to use a computerized numerical control (CNC) milling machine.

Researchers have similarly found high levels of enthusiasm regarding AR-based learning: “users report feeling higher satisfaction, having more fun, and being more willing to repeat the AR experience” (Radu, 2014, p. 1536). AR learning often requires students to “interpret data and make an argument based on evidence … as they design, test, and build their final creation” (Bartholomew, 2017, p. 27).

Once considered a technology trend of the future, these immersive tools now offer ways of interacting with the world that were not previously possible. They have the potential for individuals to share and respond to each other’s cultures and environments. As such, they offer a natural application for changemakers (Whewell et al., 2021; Habak et al., 2021).

### The changemaker movement

The changemaker movement seeks to build the skills and attributes for individuals to find innovative solutions to society’s challenges. Changemaking can thus be defined as a process of designing, refining, implementing and evaluating an innovation (Thorogood et al., 2018). Whilst changemaker skills are not necessarily new, they are contemporary, as there is “an expectation for young people to be the social leaders and innovators of tomorrow” (Alden-Rivers, Armellini, & Nie, 2015b, p. 11).

Many researchers recognise the complex nature of changemaking, highlighting the ability to identify an issue and take positive action as one of an array of relevant attributes (Alden-Rivers, Armellini, & Nie, 2015b; Thorogood et al., 2018). In addition, changemaking activities promote the development of ‘soft skills’ that are not easily taught, such as project management, persuasion, teamwork and leadership.

Strong links are made in the literature between changemaking and employability, although it is noted that changemaking places a greater emphasis on empathy, creativity and reflection (Alden-Rivers, Armellini, & Nie, 2015b, p. 3). The studies cited above emphasise the combination of empathy for others with the motivation to take creative action to solve problems, recognising that these abilities are favoured by employers. At the heart of the changemaking process is “the active, engaged student who does not passively consume knowledge but who is active in creating it” (Thorogood et al., 2018, p. 544).

### Learning theories

The learning theories of constructionism and connectivism acknowledge the role of digital technologies in knowledge construction and ways of knowing. Within our projects these theories combine to frame the development of digital literacy, cultural competence and changemaking.

#### Constructionism

The foundations of constructionism lie in the work of Seymour Papert at Massachusetts Institute of Technology, who studied how children learn and express their place in the world through interaction, computational thinking, and inquiry (Papert, 1980). Papert’s constructionist ideas take Piaget’s constructivist theories about learners actively building knowledge a stage further by suggesting that making an external, shareable product creates an ideal scenario for learning (Harel, 1990).

The process of constructing something meaningful creates conditions for building new knowledge as it makes space for the iterative development of ideas (Papert, 1980). Furthermore, Papert suggested that digital technologies could provide a constructionist environment through which learners could apply skills of inquiry and creativity to represent knowledge in various ways, anticipating the current rise of the maker movement and of computer science in schools. Social constructionists further contend that meaning arises from social processes rather than individuals (May & Mumby, 2004, Ch. 3).

As Bruner puts it, “It is this that leads me to emphasise not only discovery and invention but the importance of negotiating and sharing – in a word of joint culture creating” (Bruner., 1986, p. 127). The social context is central to the DLAB and TalkTech projects as students collaborate on the production of shareable outputs and jointly pursue questions and solutions with real world relevance.

The digital products created within the two projects therefore become both *objects to think with* (Papert, 1980) and *objects to share with others* (Kafai & Burke, 2013). Learning and knowledge construction take place through social interactions mediated by the technology tools and tangible outputs as the university students collaborate across the various countries involved in the projects.

#### Connectivism

Connectivism similarly recognises the key role of technologies in facilitating learning. It also places an emphasis on the social and cultural learning context, facilitated by technologies enabling information exchanges. In Siemens (2005) and Downes’ view (2010), connectivism promotes learning across peer online networks as students seek out and share information using internet technologies, forming a global community of learners. As Downes states, “a goal of connectivism is to facilitate the conversation and interaction around episodic learning events in a distributed environment” (Downes, 2010, p. 31). Learning in a connectivist model is cyclical, as leaners connect and reconnect with each other and with technology to find and share new information. The nature of knowledge itself shifts as new patterns of connections are constantly formed and new ideas are co-constructed through social interactions.

Two understandings therefore guide connectivism. Firstly, decisions are based on rapidly changing foundations; and secondly, the ability to see patterns and draw distinctions between important and unimportant information is vital. As a result, connectivism requires more than just networked technology to connect students for learning. Students encourage each other to learn as they tackle new problems, making use of the tools available to them: the internet, their own connections and experiences, and problem-solving skills. Learning is thus a continuous process where real life experiences and professional opportunities inform knowledge and understanding. Learners “continue to search for and gain knowledge outside of traditional education channels, such as job skills, networking, experience, and access to information, by making use of new technology tools” (Radianti et al., 2020; Siemens, 2005).

Autonomy and authenticity are key features of the changemaking activities that took place in the DLAB and TalkTech projects, as students’ personal values guided the ability to influence positive social change (Whewell et al., 2021). In line with connectivist ideas, technology supported changemaking through the ability to connect globally using internet-based communication and collaboration tools. This enabled users to share ideas and media in ways that called others to action and jointly construct new understandings. Changemaking was thus enacted through a process of “collaborative social innovation” (Toivonen, 2013, p. 2).

### Design thinking

**Fig. 4:**
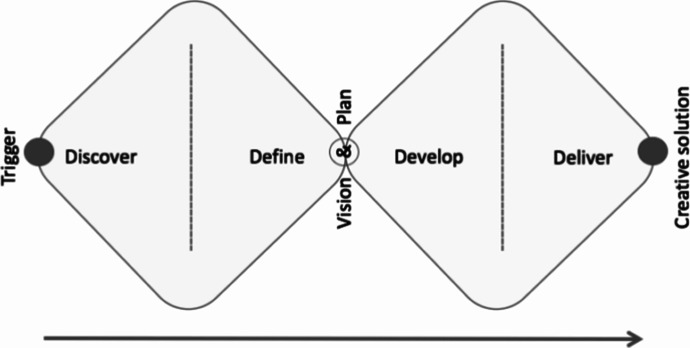
Adaptation of the UK Design Council’s ‘Double Diamond’ model (EMBL-EBI, 2021)

Both projects drew from design thinking models. Design thinking is a participatory research methodology that allows all members, including researchers and participants, to be equal and involved (Seidel et al., 2020). It also offers a structured approach to collaborative problem solving. The projects implemented a double diamond design thinking model that combines episodes of divergent and convergent thinking as students worked together to define a problem and create a solution using the immersive technologies. Figure 4 illustrates the two phases of divergent and convergent thinking as the collaboration process moves from design to delivery. The premise of design thinking is to begin with a problem to solve (Trigger).

In the DLAB project the problem was the link between video gaming and physical inactivity in school pupils, and the solution to be explored was the idea of exergaming. The term ‘exergaming’ has been used to refer to gaming which uses controllers that are moved with large bodily movements (Faric et al., 2019). In the context of this study the definition is broadened to include a range of technologies supporting physical activity. In the TalkTech project, the problem to be solved was how members of global entrepreneurial teams might present products and services to customers. Participants then offer a range of ideas and solutions in the divergent phase (Discover and Define). Ideas are brought together and shared in the Vision and Plan phase. Ideas are refined and developed over the Develop and Discover phase, converging at the Creative Solution point. This model is commensurate with the changemaker ethos of supporting autonomy and innovation.

The two projects came together in their evaluative stages to explore how university students’ cultural competencies and changemaker identities might be facilitated by technologies across the two disciplines of education and entrepreneurship. Data is elicited from a reflective questionnaire administered at the end of a project year.

### Student engagement

The use of interactive digital media has become a popular approach to engaging students while teaching digital skills (Herrington, Oliver & Reeves, 2002). Brown and Green (2016) found that the majority of VR uses in higher education are related to science, followed by humanities, arts, engineering, and social sciences. The main purpose of using VR has been for explaining a topic of interest as well as providing additional information, while the main advantages for introducing VR include increased learning, motivation, interaction, collaboration, and student engagement.

Placing students in the role of content creators promotes student understanding and engages students as they learn from one another. “Children learn in an interactive social relationship and then internalize what they learn from that relationship until they are able to function independently” (Scruggs, p. 54). Learner autonomy is a key attribute of the changemaker movement.

Rob and Rob (2018) find that constructionist learning models promote student learning and engagement. In a constructionist learning scenario, a teacher presents a problem to be solved and guides students in creating an artefact that reflects their individual learning. The use of the design thinking model in this study promotes the idea of sharing and considering multiple outcomes, reflecting the importance of learning and co-creating together.

## Method

This study employs an interpretive methodology, seeking to understand the ‘self-interpreting’ nature of human beings (Weaver & Olson, 2006). Greenwalt (2008) notes that this methodology invites participants to ‘dwell’ on their experiences and to use “as much concrete detail and context as possible” (Greenwalt, 2008, p. 391). Furthermore, the interpretive approach focuses on behaviour with meaning and intention; it stems from the individual and then tries to interpret the meaning (van Manen, 2016). This study aimed to yield data that is inductive and descriptive using a questionnaire designed to gather the experiences and reflections of its participants, and the interpretations they attached to them.

Participants were asked to complete an online questionnaire at the end of their project year that invited them to reflect upon their participation in the projects and to evaluate their skills, knowledge and attributes before and after the projects. The questionnaire had three main foci, technology skills, changemaking and cultural competences. Drawing from the changemaker attributes defined by Alden-Rivers, Armellini, and Nie (2015b), questions required participants to reflect upon the personal, interpersonal and digital skills they developed through participation in the project, the challenges and benefits of international collaboration, and their understanding of changemaking.

### Research sample

The data is drawn from the survey responses of 63 out of 85 university students participating in both projects during the 2020–2021 academic year. TalkTech students were enrolled in universities in the United States and Romania, while DLAB students attended universities in Spain, Belgium, Norway, Denmark and England. Their ages range from 18 to 35. The overall sample is purposive in that students were selected to participate in the projects due to their interest and expertise in digital technologies and changemaking. The 23 students who did not undertake the survey chose not to do so. Ethical approval was granted by all participating university ethics committees.

### Data analysis

**Fig. 5:**
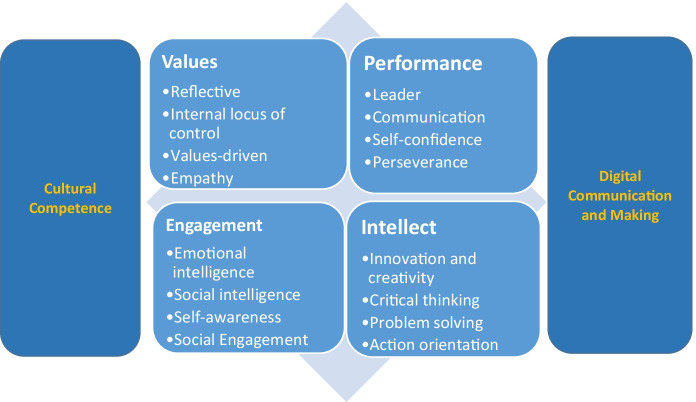
Four stranded concept of graduate identity in the context of the projects adapted from Hinchliffe and Jolly (2011) and Alden-Rivers, Armellini, and Nie (2015b)

The analytical approach depends largely on the purposes of the research and should be a key determinant in the design of the study (Kivunja & Kuyini, 2017). Bearing this in mind, a thematic analysis was undertaken based on responses to a number of open and closed questions within the student questionnaires. Firstly, we sought to establish student engagement with the digital technologies used for communicating and making within an international context. Secondly, we investigated the role of the technologies in developing students’ changemaker identities and cultural competence. The analysis was based on a process of deductive thematic coding (Clarke & Braun, 2017). Codes were drawn from the work by Alden-Rivers, Armellini, and Nie (2015b) on changemaker attributes to provide a set of key words to assign to the data. These were grouped into overarching concepts identified in Hinchliffe and Jolly’s (2011) adaptation of Holmes’ (2001) work on graduate identity, encompassing Values, Intellect, Social engagement and Performance (see Fig. 5). This approach provides a model or example of a framework for international projects. It uses existing theoretical frameworks combined in a new way, namely changemaker attributes and graduate identity.

All decision making throughout the data analysis was based on discussions between the four project researchers, who undertook an iterative process of coding to identify responses related to the research questions ensuring consistency and shared understanding of data findings (Strauss & Corbin, 1998).

### Limitations

Covid-19 has highlighted the need to explore ways to collaborate in technology enhanced and meaningful ways in international contexts. However, the distinct set of circumstances, individuals and technological affordances of this study limit its replicability. Although the findings are limited to the context of the projects, there are generalisations that can be applied to digital making, cultural competence and changemaking.

## Results

The findings of this project firstly involve student use of technologies and secondly present the development of students’ changemaking attributes and identity.

### Use of technologies

Regarding the use of technologies prior to their involvement in the projects, students reported that they had rarely used AR and VR tools and that their experience with video conferencing, collaborative writing, online photo albums, online games and blogging was limited. However, over half of the students were using mobile phones, search engines, messaging apps and social media daily. Over half thought that their ‘tech savviness’ had improved because of the project and a third felt that this was an important skill to have developed.

Students reported that their technology use changed over the course of the projects. Their responses suggest that they found the AR and VR tools exciting and easy to use. For example,

‘I learned how to create an AR and VR application. Though I'd used both of them in the past, I had no idea it could be so simple to create these forms of technology’ (DLAB)

‘It is possible to create VR and AR on your own to create really cool content. It is best to plan ahead to see which type of technologies would be most appropriate for certain situations’ (TalkTech).

Students identified uses for AR and VR across educational and entrepreneurial contexts:

‘Augmented reality can help enhance products. Virtual reality can also be applied in almost any industry.’ (TalkTech).

‘It is a technology easy to use and very helpful in many areas, especially in education’ (DLAB).

Furthermore, it was clear that students relished the opportunity to use AR and VR as creative media:

‘We had the opportunity to create amazing experiences in both AR and VR domains’ (TalkTech)

‘I loved the fact that we were free to explore tools for VR and AR and be creative’ (DLAB)

### Changemaker attributes and identity

Our set of codes and concepts are drawn from Alden-Rivers, Armellini, and Nie’s (2015b) work on changemaker attributes and from Hinchliffe and Jolly’s (2011) work on graduate identity. This is a four-stranded concept of identity that comprises Values, Intellect, Engagement and Performance (Hinchliffe & Jolly, 2011), building upon Holmes’ (2001) premise of graduate identity as being malleable and plastic. This model recognises ‘traditional’ graduate employability skills and attributes such as IT skills, interpersonal skills and work experience, but goes further to suggest that global awareness, environmental awareness and cultural awareness are increasingly important. The model effectively complements the range of changemaker attributes offered by Alden-Rivers, Armellini, and Nie (2015b) through its emphasis on diversity, cultural awareness and social responsibility (Hinchliffe & Jolly, 2011, p. 13). The Values concept comprises ‘personal ethics, social values and contextual, organisational values, including the value of entrepreneurship’ (Hinchliffe & Jolly, 2011, p. 13). The Intellect concept refers to cognitive skills, critical thinking and sector specific problem-solving skills. Performance is deemed to be the ability to learn quickly, adapt and apply skills in a new setting. Engagement is seen to be outward looking, optimistic and ‘able to meet personal, employment and social challenges’ (Hinchliffe & Jolly, 2011, p. 17).

Figure 5 represents a combination model that identifies the ways in which Alden-Rivers, Armellini, and Nie (2015b) changemaker attributes can be aligned with Hinchliffe and Jolly’s (2011) features of graduate employability. The process of grouping these developed themes that facilitated the coding and analysis of the students’ comments. The changemaker attributes demonstrated by the participants of the two projects were aligned with the graduate identity model to demonstrate ways in which technologies contributed to students’ identities as a changemakers in the twin spheres of education and entrepreneurship. This alignment helps to reinforce the link between changemaker behaviours and changemaker identities. For example, attributes such as resilience and self-esteem are aligned with values and engagement. Furthermore, if individuals are to make a difference in an international context the attributes of digital competence and internationalism are fundamental prerequisites for success.

The following section shares a selection of responses from DLAB and TalkTech participants grouped into the four overarching concepts: Values, Intellect, Engagement and Performance (Hinchliffe & Jolly, 2011) aligned with the codes drawn from Alden-Rivers, Armellini, and Nie (2015b). The DLAB participants are reflecting on the potential for technology supported changemaking in an educational context and the TalkTech participants are thinking about its application to business and entrepreneurship.

#### Values

Within the concept of Values, students showed initiative and were resourceful and self-directed in solving technology issues (Table 1).

**Table 1:** Example responses from DLAB and TalkTech participants related to the concept of values

Value	Reflective Internal locus of control Values-driven Empathy		
‘Not only did children learn about becoming a change-maker and immersive technology, they learned that technology can be used to learn about cultural differences and encourage open mindedness to lives that are different to their own.’ (DLAB)	‘This project was very different which is something I liked. It was cool to see how we could apply this technology to the real world and actually make it useful.’ (TalkTech)	‘The use of immersive technologies live across countries was really impactful as children expressed their excitement about the project, being creative and becoming changemakers.’ (DLAB)	‘A changemaker is a person who is constantly evolving so being a changemaker is a set of mind and you have to be a person who identifies problems and wants to create solutions to them. ‘(DLAB)

Participants demonstrated empathy in evaluating the impact of the collaborations on others, and they reflected on their own learning and on how others learn, demonstrating the personal and professional impact of their collaborations as educators and entrepreneurs. It was clear that exposure to international collaboration created conditions in which changemaker attributes such as empathy and reflexivity could flourish (Whewell et al., 2021).

‘The team was awesome. I liked working with people from another region or continent. You make new friends you can change thoughts.’ (TalkTech)

‘The Romanian VR did not exactly match with the concept of retail in our AR; however, we overcame this by attributing it to a cultural difference and explored why retail was perceived in this way’ (TalkTech).

This echoes Hinchliffe and Jolly’s (2011) definition of values and suggests that participants are critiquing, questioning and engaging in critical dialogue (Whewell et al., 2020). Hinchliffe and Jolly (2011, pp.13–14) refer to “personal ethics, social values and contextual, organisational values, including the value of entrepreneurship”. The idea that social values encompass social responsibility and an awareness of cultural diversity links with changemaker attributes.

#### Intellect

Within the concept of Intellect students were action-orientated as they planned to use AR and VR technologies in their workplaces (Table 2).

**Table 2:** Example responses from DLAB and TalkTech participants related to the concept of intellect

Intellect	Innovation and creativity Critical thinking Problem solving Action orientation	
‘Creative apps such as AR and VR can be the answer for a brilliant start up.’ (TalkTech)	‘I have learned how to use a myriad of tools that I had never considered using before and I have found ways to integrate this into the classroom to support learning.’ (DLAB)	‘In order to become changemakers we need to reach beyond ourselves’ (DLAB)

For example, during the coronavirus outbreak Belgian students used their Mind and Makerspace to make gowns and masks for care providers. Similarly, students in Romania showed a creative vision for real world applications of VR and AR:

‘Given that I had the area of retail, I learned that AR and VR are the future of this industry’.

The authenticity of the digital making was important to them,

‘It was cool to see how we could apply this technology to the real world and actually make it useful’.

Both groups recognised the way in which collaboration amplified the creative problem-solving process,

‘...combine ideas to have a better idea’, ‘such cool ideas came from these (mind mapping)’.

#### Engagement

Within the Engagement concept students were self-aware and showed a growth mindset, understanding that personal attributes are not fixed and can be developed (Table 3).

**Table 3:** Example responses from DLAB and TalkTech participants related to the concept of engagement

Engagement	Emotional intelligence Social intelligence Self-awareness Social engagement	
‘I have learned that children are very innovative when it comes to approaching projects such as this and bring interesting insights based on their experiences - many which differ from my own.’ (DLAB)	‘Working hard about something I am passionate, breaking boundaries, going outside of normality, using creativity to teach’ (DLAB)	‘The importance of carrying out projects at the international level to improve the field of interculturality.’ (DLAB)

They displayed emotional intelligence by acknowledging difficulties and overcoming negative feelings, and social intelligence by appreciating diversity and learning from others.

‘I learned that communicating can sometimes be difficult which makes international business endeavours complicated’

‘A changemaker is a person who is constantly evolving so being a changemaker is a set of mind’

‘We have been able to learn from each other’.

#### Performance

The Performance concept encompasses the attributes of confidence, perseverance and drive as students tried different communication strategies to accommodate language and cultural differences and tested alternative solutions to solve problems (Table 4):

**Table 4:** Example responses from DLAB and TalkTech participants related to the concept of performance

Performance	Leader Communication Self-confidence Perseverance		
‘Learning how to effectively communicate with people in a different time zone and who you don’t have face to face contact with was very good.’ (TalkTech)	‘I learned how to deal with time zone differences while collaborating, and how to speak more effectively with ESOL students.’ (TalkTech) It took some explaining to get the time difference and language barrier, as well as varying lifestyles and schedules (TalkTech)	I learned how to work together on something big. We organised two international days with five countries while divided by hundreds of miles. It seemed like a normal thing to do but looking back I’m really impressed.’ (DLAB)	‘Communication was a large factor in working on this project and getting experience communicating with international students was a learning experience I valued greatly.’ (TalkTech)

‘We were unable to complete the project as intended however I think we did well to overcome this and complete the project digitally.’

Communication and digital literacy also sit within this concept and, alongside the international context, these were key attributes to both projects. Students valued the opportunity to communicate internationally and the use of technologies created conditions that supported the development of cultural competence,

‘The most important thing I’ve learned is communication between two vastly different groups’,

‘Working with people you’re not used to and who present different working methods, this is overcome through communication and dialogue.’

‘I have learned how technology can be used for trans-cultural learning’.

The impact of the international collaboration on leadership skills was clear as students focused on applying their new AR and VR skills to their future careers in entrepreneurship and education,

‘It’s the future’, ‘it could give a unique and creative view of the business’

‘I can share this knowledge with future colleagues to also improve their teaching’

Through their changemaker activities, students showed a drive to pursue global citizenship, and an appreciation of cultural diversity and of culture’s contribution to sustainable development that is very much aligned with SDG 4 Target 4.7.

## Discussion and conclusions

The aim of this study was to explore ways in which digital technologies could help university students develop changemaker attributes and identities in international contexts. We wanted to show how this was demonstrated in practice through the sharing of digital artefacts drawn from immersive technologies within a connected community that provided an authentic context.

Both connectivism and constructionism highlight the importance of building social relationships between learners in a learning context. Such relationships are vital to developing changemaker attributes and identities. Changemakers also must build social connections with colleagues to become effective team leaders, creative problem solvers, and proactive solution innovators. Creating immersive environments to experience new places and ideas as part of international projects motivates students by providing an opportunity to develop skills grounded in these learning theories. Design thinking encourages collaborative problem solving to create a common solution. Inherent in the process of collaboration is the need for learners to interact effectively with each other. Communication is a key aspect of both the DLAB and TalkTech projects. Consistent with connectivist and constructionist learning theories, students use internet-based tools to create social relationships as well as digital media artefacts.

Our findings indicate that the students involved in the DLAB and TalkTech projects gained changemaker skills, attributes, and identities as changemakers.

### The role of constructionism

Firstly, in line with constructionist theory, this paper highlights the role of digital making in achieving our aims. By co-creating and sharing original immersive environments and exergames created through AR and VR apps, university students empathised with each other’s realities and cultural norms. The making and sharing of digital artefacts within a design thinking framework supported students in developing understanding of the diversity of opinions and their various cultural contexts. The digital tools not only allowed students to connect effectively to each other, but to apply their ideas to real world educational and entrepreneurial contexts.

### The role of connectivism

Secondly, internet technologies provided a connectivist environment that facilitated effective international collaboration within a social innovation community (Toivonen, 2013). Students interacted with each other across time zones, overcoming language and technology barriers to create tangible outputs that reflected their worlds. In doing so, they developed cultural competence by demonstrating skills of empathy, resilience, innovation, and creativity, all of which are attributes of changemakers necessary to thrive as educators or entrepreneurs in a global economy (Alden-Rivers, Armellini, & Nie, 2015b; Whewell et al., 2021). The international collaboration thus provided opportunities to develop the ability to empathise and interact effectively with people from other cultures. This ties in with Li′s recent examination of the relationship between two major constructs of cultural competence: cultural intelligence and intercultural competency (Li, 2020). Our findings suggest that technology-supported changemaking can influence both aspects of cultural competence across educational and entrepreneurial contexts.

### Combining constructionism and connectivism

Students drew upon skills of tolerance, respect, communication and team work, as well as knowledge specific to the fields of education and entrepreneurship (Alden-Rivers, Armellini, & Nie, 2015b). The use of immersive technologies added real-time authenticity to the challenge to make digital artefacts aimed at effecting change whilst demonstrating cultural sensitivity and innovation. It was clear that students placed value not only on the digital artifacts created (the products) but also on the international collaboration (the process). In this way, the combination of immersive technologies and internet technologies created an environment for co-constructing new knowledge (Papert, 1980).

### Conclusions

Digital tools and the internet made possible the collaborations described here. Students who master collaborative writing and content curation, synchronous and asynchronous communication tools, file sharing and multimedia tools have the skills to work in a global economy. And, as our projects demonstrate, VR and AR add another dimension to learning, enabling students to create and share immersive experiences with each other at a distance, placing themselves in contexts only otherwise imaginable. The ability to use technologies has thus become a key component of changemaking in an international context.

Many of the technologies used in this study have been available for a significant amount of time; others have recently been gaining pace. However, their combination in this context has demonstrated potential for changemaking. We have identified a role for immersive technologies to engage students to bring their own skills, cultures, and experiences to real time authentic projects. Co-constructing and sharing digital artefacts provides an effective environment for changemaking endeavors. Working with multiple tools including AR and VR, communicating online and planning together improves students’ communication, critical thinking, innovation and group working across both educational and entrepreneurship scenarios.

A twenty-first century graduate needs global mindfulness, self-efficacy and digital literacy within their skills repertoire to contribute effectively to society. The link between authentic real-world issues motivating changemaking is key to creating an environment in which students can develop and apply these skills. We have seen that immersive technologies can become a platform for the development of cultural competences through the process and the products of digital making in an international context, and that they can foster empathetic, values-driven and action orientated individuals.

This paper concludes that the combination of an international context and the use of immersive technologies, together with real world educational or entrepreneurial challenges offers students an environment in which they can hone changemaking skills and develop cultural competences. Increasingly, employers recognise the need for employees who are culturally and digitally literate with the skills to enact change. The process of connecting and co-creating as digital makers provides university students with a productive arena to make decisions, solve problems, and effect solutions as changemakers.

We recommend that future researchers seek to decipher the dynamics of collaboration and changemaking by tracking the flow of ideas within digital communities of innovation.
